# Water quality drives the regional patterns of an algal metacommunity in interconnected lakes

**DOI:** 10.1038/s41598-021-93178-9

**Published:** 2021-06-30

**Authors:** Min Sung Kim, Seok Hyun Ahn, In Jae Jeong, Tae Kwon Lee

**Affiliations:** Department of Environmental Engineering, Yonsei University, Wonju, 26493 South Korea

**Keywords:** Community ecology, Microbial ecology

## Abstract

The metacommunity approach provides insights into how the biological communities are assembled along the environmental variations. The current study presents the importance of water quality on the metacommunity structure of algal communities in six river-connected lakes using long-term (8 years) monitoring datasets. Elements of metacommunity structure were analyzed to evaluate whether water quality structured the metacommunity across biogeographic regions in the riverine ecosystem. The algal community in all lakes was found to exhibit Clementsian or quasi-Clementsian structure properties such as significant turnover, grouped and species sorting indicating that the communities responded to the environmental gradient. Reciprocal averaging clearly classified the lakes into three clusters according to the geographical region in river flow (upstream, midstream, and downstream). The dispersal patterns of algal genera, including *Aulacoseira*,* Cyclotella*,* Stephanodiscus*, and *Chlamydomonas* across the regions also supported the spatial-based classification results. Although conductivity, chemical oxygen demand, and biological oxygen demand were found to be important variables (loading > |0.5|) of the entire algal community assembly, water temperature was a critical factor in water quality associated with community assembly in each geographical area. These results support the notion that the structure of algal communities is strongly associated with water quality, but the relative importance of variables in structuring algal communities differed by geological regions.

## Introduction

The metacommunity concept is an important approach for community ecology because it allows both local (e.g., nutrient, biotic interaction) and regional (e.g., dispersal) factors that contribute to community assembly to be identified^1^. Interest in community assembly is increasing because the local community is constantly reassembled in response to changes in the local environment, and the diversity and functionality of the community are controlled by the spatial distribution and interaction of species in the community^2,3^. To understand the role of the community assembly in the field, focusing on the community level (such as the metacommunity concept), rather than the species level, can provide new insights to associate the environmental factors with the community^4^.

Elements of metacommunity structure (EMS) is an useful tool that evaluates the assembly process of the community and determines the effects of environmental factors on the community assembly by assessing community patterns^5,6^. The EMS calculates three elements (coherence, turnover, and boundary clumping) to identify the idealized metacommunity pattern (e.g. checkerboard, random, evenly spaced, Gleasonian, or Clementsian pattern)^7^. The patterns facilitate the search for general rules determining metacommunity structure^6^. EMS approaches have been applied to terrestrial and aquatic systems for various organisms. Most studies on fish communities have been focused on freshwater ecosystems, and only a few studies have dealt with insects and zooplankton^8–12^. The algal community has largely been neglected when applying the EMS approach. Although the abundance of algal species is affected by water quality, such as phosphorus and nitrogen levels in the water, it has not been proven whether the assembling mechanism of algal communities is also affected by water quality.

The incidence of algal species is influenced by complex relationships between biological and environmental factors such as species dispersion, competition, water quality, and topography^13,14^. Although independent biological and environmental factors have been identified for the incidence of a single algal species in laboratory conditions, the study of factors affecting the algal community is limited to freshwater environment^15^. Relationships between these factors and the algal community have been found to vary across regions and spatial scales^16^. A few studies have researched on the algal community in the river-connected lakes, where is an aquatic system in which species disperse naturally and share many algal species. The structures of algal community are simultaneously determined by changes in environmental factors, even in the same regions^14,15^.

There are also reports of the importance of biotic interaction, such as increases in algal species, biotoxin production and changes in bacterial community in determination of algal community in previous studies^17^. As algal community change is a complex process determined by biotic and abiotic variables, understanding changes at the community level, rather than understanding individual algal species or population, can be important in responding to various environmental problems caused by algal community in the riverine ecosystem.

The goal of this study was to understand how environmental variables influenced algal community assembly in six river-connected lakes in South Korea. More specifically, we studied whether the algal metacommunity structure respond to water qualities in the river-connected lakes and how well can explained variability in the algal community-water quality relationships across the multiple lakes. The algal community was examined for the idealized metacommunity structures at each lake using long-term (8 years) monitoring datasets and how environmental factors (water qualities) in each of the EMS analyses were associated with algal community assembly in the lakes. Our present study provides new comparative information about the response of the algal community to local environmental factors, although the metacommunity structures are largely invariable at different biogeographic scales in river-connected lakes.

## Materials and methods

### Algal community sampling and data acquisition

**Figure 1:**
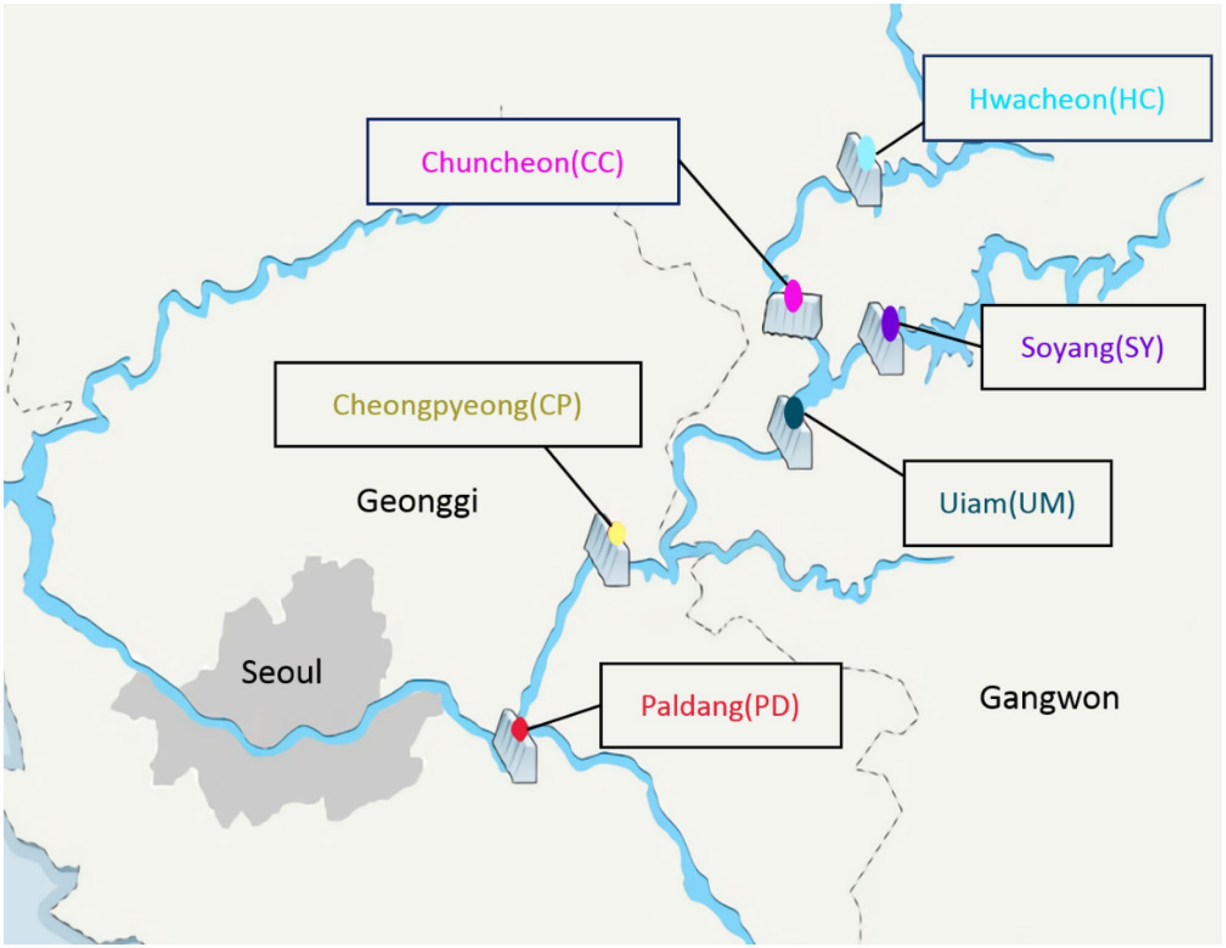
The geographical information of the six North Han River lakes. Figures were created by R 4.0.2 (https://www.R-project.org] and adjusted using Adobe Illustrator CS5 v 15.0.2 (https://www.adobe.com/products/illustrator.html).

Field samplings were performed at six lakes of the North Han River, Paldang (PD), Cheongpyeong (CP), Uiam (UM), Chuncheon (CC), Soyang (SY), and Hwacheon (HC) (Fig. 1). The North Han River (37°06′–38°06′N, 127°16′–127°49′E) located on the warm temperate zone of humid continental climate with mean annual temperature from 14.4 to 15.6 and total annual precipitation of 976–1581 mm. The monsoon season generally occurs between June to August, accounting for 73% of the annual precipitation. In detail, monthly water samples were collected using from 2008 to 2016 (except in 2014) between March to November. Integrated water samples were collected 1 m below the surface of each lake using a Van Dorn sampler (Horizontal water sampler, iStech, Korea). The nine water quality parameters including temperature, conductivity, pH, BOD (biological oxygen demand), COD (chemical oxygen demand), TN (total nitrogen), NH_4_^+^ (ammonium), TP (total phosphorus), and PO_4_^−^ (phosphate) were measured following the Korean Standard Methods for the Examination of Water^18^.

Algal samples were simultaneously collected with water samples and analyzed following the microscope counting method^19^. Algae were identified following the criteria of Hirose^18^ and Chung^18^ in the SR chamber (Graticules S52 Sedgewick Rafter Counting Chamber, Structure Probe, Inc., West Chester, USA) using an inverted microscope (Axioplan; Carl Zeiss, Germany) with 200–400× magnification. The algal cell density (unit: the number of cells per liter) was calculated by counting the number of cells in 200 grids out of a total 1000 grid in the chamber and then multiplying the result with conversion factor as 5. The dataset for algal community was pre-processed by merging species to genus level for reducing the variance of the distribution of algal population. Totally, the dataset consisted of 72 (9 months × 8 years) samples in each lake with nine chemical properties and 55 genera.

### Element of metacommunity structure analysis

EMS analysis consists of three components: coherence, turnover, and boundary clumping. Through EMS analysis, idealized metacommunity models are determined^5^, or quasi-models^6^. Coherence is assessed by counting the number of embedded absences in the ordinated matrix and comparing this to a null distribution. We identified the metacommunity structure with the presence/absence algal matrix based on the R1(fixed–fixed) model as the null model. Turnover is calculated from the number of species replacing each other from site to site^5^. Boundary clumping is evaluated by comparing the observed distribution of range boundaries with an expected equiprobable distribution^5,6^. To identify metacommunity structure at sampling sites, the coherence, turnover, and boundary clumping were computed in R^18^, using the ‘metacommunity’ function in the ‘metacom’ package (version 1.5.2). The metacommunity structure was determined using the *P*-value and z-score. When all three components of coherence, turnover, and boundary clumping had significant *P*-values, 12 metacommunity structures were identified by z-score and Morisita index. Firstly, the metacommunity structures were classified by coherence z-score into a checkboard (greater than 1.96), random (− 1.96 to 1.96), and nest or gradient type (less than − 1.96). The nest or gradient type was separated according to whether the turnover z-score was positive (nest type) or negative (gradient type). If the turnover z-score value was between − 1.96 to 1.96, the metacommunity structure became a ‘quasi-structure’ (i.e., quasi-nested, quasi-Clementsian). Lastly, Morisita index (I) separated gradient metacommunity structures as Clementsian (I > 1), Gleasonian (nonsignificant), and evenly spaced (I < 1). The metacommuntiy order of each sample was calculated by reciprocal averaging to ordinate the site-by-species matrix. Then, we ranked the site score following order of the samples in the overall metacommunity structure.

### Statistical analysis

The number of observed genera during sampling was calculated as richness. Statistical analysis was conducted using R (version 3.6.1). All variables were checked for normality with the Shapiro-Wilks normality test. If the data obeyed the normality test, ANOVA tests were performed to compare richness, site score distribution and nine water quality parameters of six lakes. Otherwise, the Kruskal–Wallis test was used. Dunnett’s rank-based multiple comparisons were performed to identify variables has the significance difference among groups by environmental variables and richness. All step computed in R using mctp function of the “nparcomp” package. The hierarchical clustering was calculated by using the presence/absence matrix and Euclidean distance of six lakes from the ‘hclust’ function in R. To find key genera of three groups which separated by the site score (High: SY and HC; Middle: CC, UM, and CP; Low: PD), the R package ‘random forest’ (version 4.6-14) was used for a random forest classification^20^. The classification model was designed with 131 trees with 1,000 permutations using sampling data and was validated by the confusion matrix method. From the classification models, six key indicator genera (top 10% of total genera) were selected based the values of mean decrease in Gini, indicating that a measure of each variable’s contribution to the impurity of the resulting RF model. High value of Mean Decrease Gini tends to have high purity to the model’s homogeneity. The ‘cca’ function of the ‘vegan’ package (version 2.5-6) was used to implement canonical correspondence analysis (CCA)^21^ to assess which environmental variables were associated with site score distribution and the underlying metacommunity structure of the North Han River. The correlation analysis was performed between CCA 1 and water quality parameters using the Pearson or Spearman method on normal or non-normal datasets, respectively.

## Results

### Diversity of the algal community

**Figure 2:**
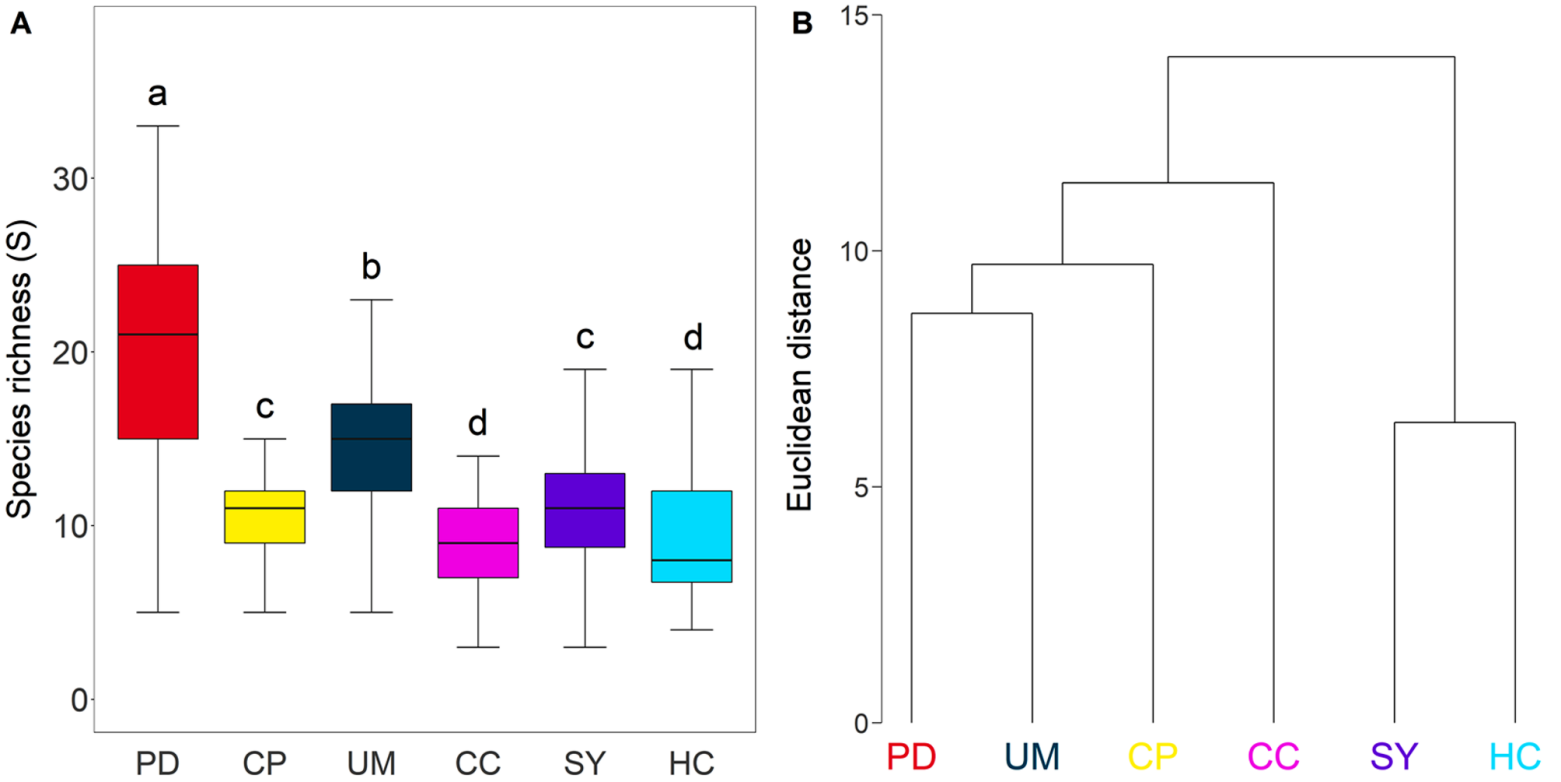
(**A**) The richness of algal community in sampling sites. (**B**) Dendrogram of the cluster analysis using presence/absence data from sampling sites using euclidean distance. The significantly difference between sampling sites marked as difference small letters. Figures were created by R 4.0.2 (https://www.R-project.org) and the labels were adjusted using Adobe Photoshop CS6 13.0.6 × 64 (https://www.adobe.com/products/photoshop.html).

In total, 55 genera were observed in the six river-connected lakes. Genus richness was highest in PD (5–33), followed by UM (5–17), SY (2–19), CP (5–17), CC (3–14), and HC (4–19) (Fig. 2A). The richness of PD and UM, where two rivers meet (Fig. 1), was significantly higher than other lakes (Kruskal Wallis test followed by Dunnett test, *P* < 0.01). CC and HC, which had the lowest richness, did not differ from each other because they share the same upstream origin. The hierarchical clustering analysis based on the beta-diversity of algal community compositions produced two groups, upstream (HC and SY) and downstream (CP, CC, UM, and PD) (Fig. 2B). As for the richness results, PD and UM were most similar in downstream groups. CP, which is regionally located between PD and UM, showed the most similar algal community composition to those lakes. These results suggest that algal diversity is highly influenced by hydrogeological factors.

### Elementary of metacommunity structure

**Figure 3:**
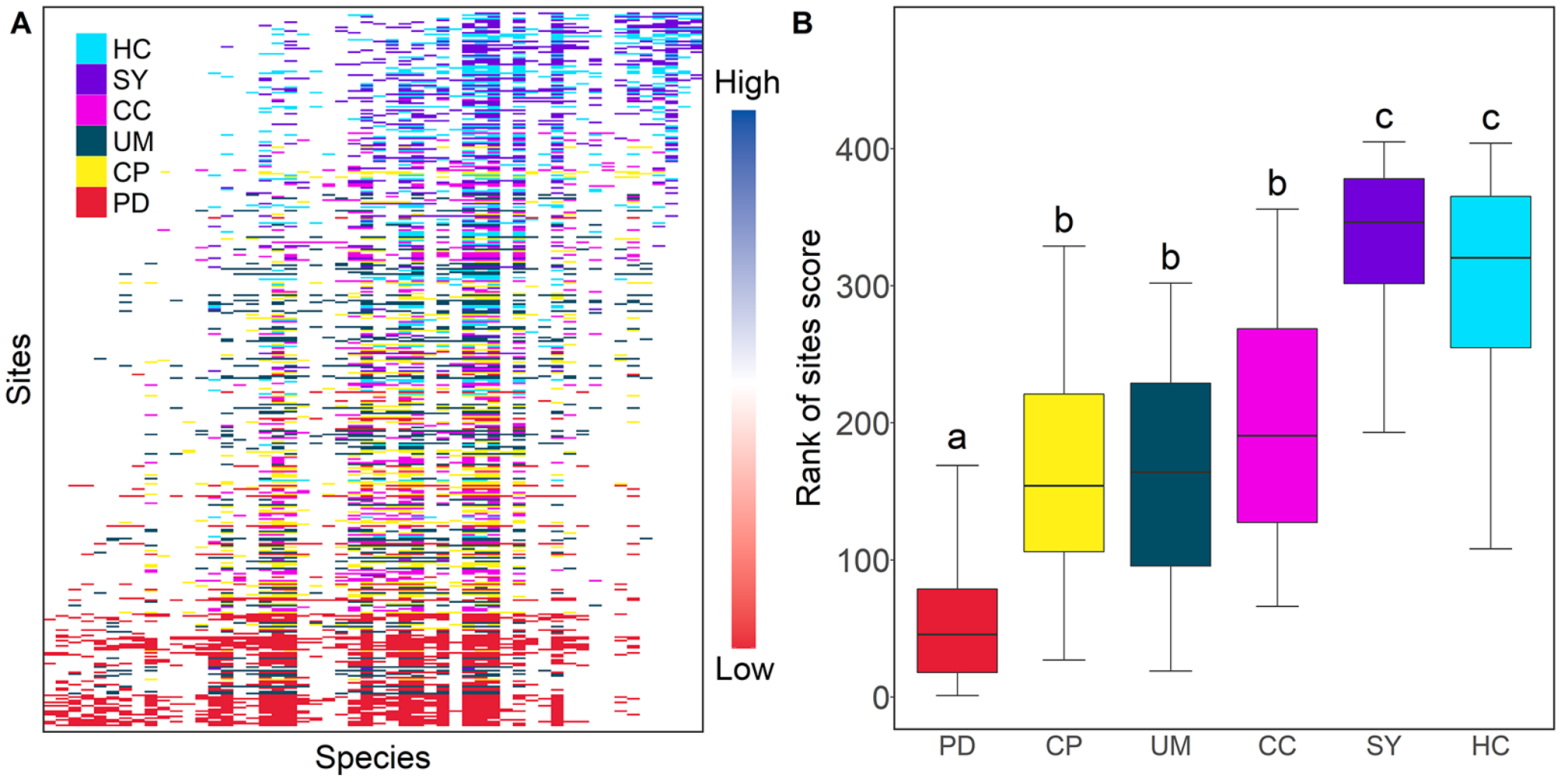
(**A**) Metacommunity structure of the North Han River and (**B**) locational distribution of each lake in entire metacommunities. The rank of site scores was determined from ordination of site in EMS structure. Different small letters indicate significant differences between values of sites score distribution. Figures were created by R 4.0.2 (https://www.R-project.org) and the labels were adjusted using Adobe Photoshop CS6 13.0.6 × 64. (https://www.adobe.com/products/photoshop.html).

Across all datasets, EMS analysis revealed positive coherence, positive turn over and large values (> 1) of boundary clumping with Clementsian structure (Table 1), with ranges of algal genera contributing most to these patterns (Fig. 3A). The Clementsian structure indicates that the community was assembled by the environmental gradient. Most individual lakes also exhibited the Clementsian structure as a best-fit pattern of metacommunity structure (Table 1, Fig S1). In contrast, PD exhibited a quasi-Clementsian structure due to its non-significance in turnover. Even though all lakes were analyzed by season, they were identified as Clementsian or quasi- Clementsian as the same as the abovementioned results (Table S1). These results support the algal communities were strongly associated with environmental factors regardless of variation in biogeographic units or seasons.

**Table 1:** Result of coherence, species, turnover, and boundary clumping for algal communities from the sampling sites.

	PD	CP	UM	CC	SY	HC	All
**Coherence**							
Abs	1317	1004	1209	940	1032	1169	11,336
Z-score	11.7	12.2	13.3	11.8	14.7	9.8	39
*P*	0.001	0.001	0.001	0.001	0.001	0.001	0.001
Sim mean	1561	1382	1661	1348	1464	1524	15,589
Sim SD	21	31	34	35	29	36	109
**Turnover**							
Rep	32,361	59,527	63,792	37,806	58,632	46,457	1,995,007
Z-score	− 1.8	− 5.4	− 4	− 2.2	− 2.9	− 4.3	− 5.5
*P*	0.08	0.001	0.001	0.028	0.004	0.001	0.001
Sim mean	21,652	24,895	32,923	25,007	34,452	22,428	895,529
Sim SD	6050	6440	7817	5819	8345	5596	198,657
**Boundary clumping**							
Index	2.4	2.5	2.2	2.2	3	2.1	9.5
*P*	0.001	0.001	0.001	0.001	0.001	0.001	0.001
*df*	65	64	65	63	65	65	402
Metacommunity structure	Quasi-Clementsian	Clementsian	Clementsian	Clementsian	Clementsian	Clementsian	Clementsian

Abs, number of absences; Rep, number of replacement; Index, Morisita’s index.

**Figure 4:**
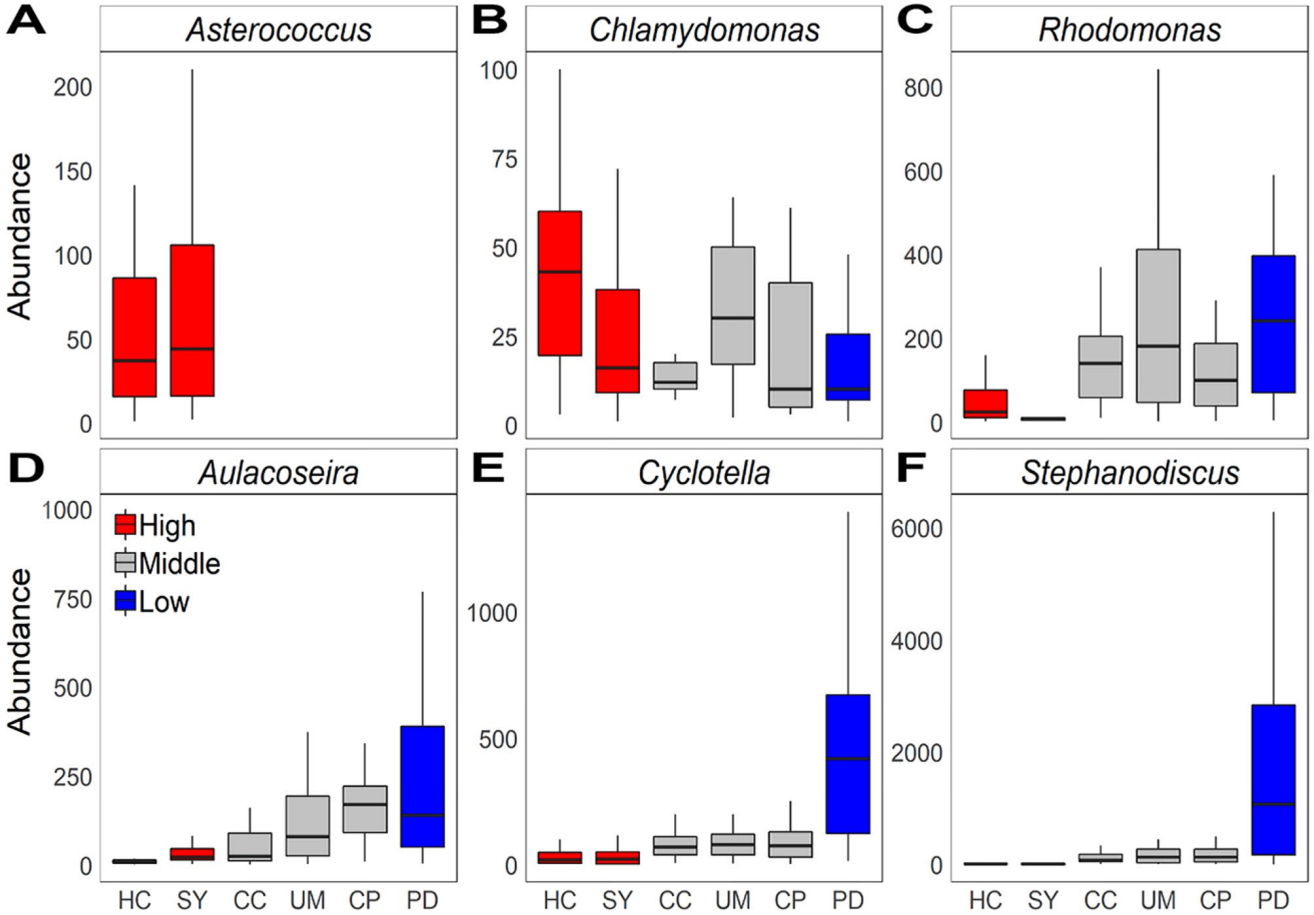
The abundance distribution of key indicator genera (**A**) *Asterococcus*, (**B**) *Chlamydomonas*, (C) *Rhodomonas*, (D) *Aulacoseira*, (E) *Cyclotella* and (F) *Stephanodicus*. The genera with the top 10% mean decrease Gini value was selected as key indicator genera. Figures were created by R 4.0.2 (https://www.R-project.org) and the labels were adjusted using Adobe Photoshop CS6 13.0.6 × 64 (https://www.adobe.com/products/photoshop.html).

The Clementsian structure across all datasets had three compartment-by-site scores, which were determined by an EMS ordination procedure for ordering algal genera and sites (Fig. 3B). CP, UM, and CC, located midstream, were between 100 and 300 in the rank of site scores, and significantly different from upstream (SY and HC) and downstream (PD) (Kruskal Wallis test followed by Dunnett test, *P* < 0.05). These results are differentiated from the beta-diversity results, which were divided into two groups. Although genera dispersed through river hydrologic connections and six lakes shared considerable numbers of genera, the occurrence of some genera was unique geographically. The indicator analysis confirmed that the abundances of key indicator genera were clearly varied depending on the location of river networks (Fig. 4). The distribution of *Aulacoseira, Cyclotella*, and *Stephanodiscus* increased proportionally from upstream to downstream, while *Chlamydomonas* decreased. The distribution of *Asterococcus* was unique to the upstream group. These results support the contention that metacommunity analysis is capable of analyzing the assembly of algal communities in detail at the community level as well as at the genus level. Lakes located downstream could be classified into two additional clusters (down: PD, and mid: CP, UM, and CC) according to the assembly characteristics.

### Environmental drivers

The water quality of all lakes is summarized in Table S2. Although clear regional differences were not observed in algal diversity and EMS results, the water quality of the downstream (PD) and upstream (SY and HC) groups was statistically different, except for pH. The midstream (CP, UM, and CC) varied regionally within the range of water quality values between upstream and downstream, while COD and TN gradually increased from downstream to upstream. Seasonal or annual temperatures and precipitation, which are known to be important for algal growth, could not be seen as significant differences between lakes (Table S3 and S4).

To identify the environmental drivers of algal community, the association between water quality and site scores generated from EMS analysis was evaluated. The CCA axes were defined by reciprocal averaging, which is the same ordination method used to identify the main gradient of algal distribution in the EMS framework^4^. Site score and CCA axes were highly correlated (Spearman's ρ = 0.92, *P* < 0.001), indicating that axes from both analyses represent variations in the same latent environmental gradients. The variation in temperature was most highly associated with the CCA1 axes along with the metacommunities structure in each location (Table 2), whereas temperature was not important in all lakes (loading = − 0.061). Temperature was positively related to the EMS ordination axes of upstream (loading = 0.893), but negatively related to axes of downstream (loading = − 0.801) and midstream (loading = − 0.547). Moreover, other environmental variables which were highly associated with EMS ordination axes (i.e. loading < 0.500 or > 0.500) were significantly different by location. Conductivity and COD were the environmental variables highly associated with ordination axes for upstream, but pH or BOD were associated downstream and midstream. Compared to the individual analyses of lakes, these results tended to be similar (Table S5).

**Table 2:** Contribution of environmental variables in first axis of canonical correspondence analysis (CCA).

	ALL	Up	Mid	Down
Temperature (℃)	− 0.061	**0.893**	− **0.547**	− **0.801**
Conductivity (μS/cm)	**0.743**	**0.546**	0.237	0.408
pH	0.354	0.308	0.413	**0.625**
BOD	**0.57**	0.138	**0.598**	**0.659**
COD	**0.711**	**0.655**	0.214	0.361
TN	0.355	0.245	0.193	0.168
NH_4_^+^	0.072	0.102	0.234	0.125
TP	0.189	0.219	− 0.027	− 0.095
PO_4_^−^	0.012	0.481	− 0.155	− 0.257

Each value indicated loading value of first axis in CCA using corresponding algal community and environmental variables data according to groups.

The bold values mean highly environmental variables highly correspond (< 0.5) to first axis of CCA.

BOD, Biochemical oxygen demand; COD, Chemical oxygen demand; TN, Total nitrogen; NH_4_^+^, Ammonium; TP, Total phosphorus; PO_4_^−^, Phosphate (unit: mg/L).

## Discussion

We used EMS analysis combined with CCA to identify the relationship between water quality and assembly of algal communities in river-connected lakes. Most of the algal metacommunities for each lake follow a Clementsian structure, characterized by a continual change in algal composition at the genus level along environmental gradients. EMS produced three regional compartments (upstream, midstream, and downstream) by reciprocal averaging score. CCA revealed that three compartments were associated with different variables of water quality. Therefore, algal communities along the river were generally assembled depending on the water quality of the region, even though algal communities were dispersed and genera were shared through hydrological connections.

The EMS and the conventional diversity approach were compared to understand the importance of biogeological features on the algal community in river-connected lakes. The alpha diversity (richness) varied significantly depending on individual lakes (Fig. 2A), but the beta diversity and EMS approach could provide clear clustering by biogeographical features (Figs. 2B and 3B). Since beta diversity measures the changes in diversity of species from one site to another^22^, beta diversity should provide similar clustering results to the EMS approach. Nevertheless, it is worth noting that the number of regional partitions was different in the two approaches. Because EMS is based on site-by-species incidence, matrices consider whether the community responds to environmental gradients by measuring the proportional species turnover^1^, thus the EMS approach could provide discriminatory information compared to beta diversity.

Metacommunities in biogeographical regions or individual lakes were either Clementsian or quasi-Clementsian (Table 1 and S1). Clementsian structures arise when communities are actually changing consistently through groups of species that respond in a similar way to environmental gradients^23^. These results are consistent with the previous reported that metacommunity structure of diatoms showed Clementsian in river-connected mountain stream^24^. According to the river continuum concept (RCC), the physical properties of the riverine ecosystem were determined by flowing water from upstream to downstream. The physical properties changed the chemical system and biological communities in the responded or induced environmental gradient^25^. Synchronous species turnover is a phenomenon that occurs in ecosystems that share a significant proportion of species^26^. Clementsian structure is not rare, and they have already been reported for other aquatic organisms^9,27^. Most species found in riverine ecosystems are generally regulated by species dispersal and sorting^13^, so that the downstream sites shared high proportions of genera, while upstream sites showed significant differences in this study (Fig. 3B and Fig. S2).

The lakes located in midstream (CP, UM, and CC) shared highly similar distributions of genera but were significantly different from downstream (PD). PD, at the confluence of three rivers, is prone to dispersal of other genera from other rivers. These partially explain the quasi-Clementsian structure and distinct patterns compared to midstream.

One of the advantages of the EMS approach is identifying the environmental variables that influence community assembly by correlating reciprocal averaging and environmental variables. CCA, which is based on reciprocal averaging and multiple regression, was used to determine which environmental variables were associated with gradients along which metacommunities were structured^21^. The algal communities across broad geographical gradients showed consistent Clementsian structure. Clementsian structure emphasizes discrete ‘community types’ along ecological gradients, such that subgroups of species replace other subgroups in space^23^. Such variation also suggests that subgroups of species either respond similarly to environmental variation or are affected by similar historical effects such as drought, flood and other environmental perturbation that occurred before^28^.

Conductivity, COD, and BOD were found to be the most important variables (Loading > |0.5|) of the entire algal community assembly. Conductivity is an indicator of overall variation in water chemistry, associating with levels of nutrients such as phosphate, nitrate and ammonia. COD and BOD is the most commonly used organic pollution indicator in water bodies^29^. The amount or type of organic matter has a significant effect on microbial community structure. In this study, we also found that beta diversity of algal community varied significantly depending on the lake specific concentration of COD (Fig. 2B and Table S2). The association between COD concentration and beta diversity suggests that COD make a significant contribution to the structure of algal communities. Previous reports also identify conductivity, COD, and BOD as the main drivers of the algal community composition^14,30,31^, indicating the importance of these factors as a driver of algal composition in the rivers. This contradicts, in part, the work of Padisak et al.^32^ who found TN and TP to be important drivers of functional groups in the river, while conductivity and COD were not significantly correlated with functional groups. However, untangling these communities and analyzing each lake type classified by the EMS approach revealed that the algal community could be distinguished by presenting a different relationship with temperature. The compositions of algal communities are remarkably influenced by temperature in a single lake^15,33^. Since lakes classified through the EMS approach had a similar algal composition (Figs. 2, 3), it is possible to explain that temperature acted as an important variable, unlike the results where the entire algal community is analyzed. Besides temperature, the variables strongly related to algal communities were conductivity, COD, and BOD, which concurs with the entire algal community analysis, but that importance differs depending on the location of the lake. Depending on land use and population density along the river, the types and concentrations of organic matter flowing into rivers vary, and the algal communities, which are strongly affected by differences in organic matter, are sensitive to regional differences^34^. This may explain why environmental variables were found to regionally influence the algal community assembly in river-connected lakes.

The relationship between the main structure of the entire metacommunity and the three lake types classified by biogeographical regions (up-, mid-, and downstream) reveals the role of spatially structured factors on algal composition. Previous research on lakes has shown that geographical distance strongly influences the algal community distribution^16^. The results of the current study also provide evidences that more than half of the genera are shared regardless of the lake location as species dispersal is a main driver of community assembly in a riverine ecosystem. For example, *Stephnaodiscus* and *Cyclotella*, were reported as ‘weedy’ genera that highly dispersed and their abundances were known to be affected by level of nutrients^35–37^. Our results also showed the abundance of *Stephnaodiscus* and *Cyclotella* were closely related to the concentration of TP and COD along the river (Fig. 4 and Table S2). The lake specific genus was also observed such as *Asterococcus* in upstream (HC and SY). This unique genus has known to be associated with the presence of freshwater organisms (e.g. fish and bivalve) as well as nutrients level^35^. The ecological and limnological properties of upstream may accommodate the unique genus. The uniqueness of the algal communities in each lake supports that the assembly of algal community is affected by species sorting. These results are consistent with previous findings that algal communities are determined by species dispersal when habitats are shared in aquatic environments^13^. The EMS approach is powerful in detecting compartmentalized structures according to spatial distribution and provides a fruitful interpretation of algal communities at the species and community levels.

## Conclusion

River-connected lakes were used to address patterns and the underlying process of metacommunity organization of algal communities in freshwater. The approach based on metacommunity used ecological features, providing a fruitful starting point for more sophisticated analyses of variations in algal community structure. Our findings strongly suggest that algal metacommunities showed Clementsian structures over long spatial extents through the water body. The EMS approach combined with CCA facilitated the interpretation of the effect of environmental variables on the variation of the algal community assembly, and its effects across biogeographic regions in riverine ecosystem. In addition, the results also provide insight into biogeographical patterns of algal community structure in freshwater by comparing the beta diversity and EMS approach. This finding may also be applicable in aquatic ecosystems when studying local communities across large spatial scales.

## Supplementary Information

**Supplementary Information.**

## References

[1] Leibold MA (2004). The metacommunity concept: a framework for multi-scale community ecology. Ecol. Lett..

[2] McGill BJ, Enquist BJ, Weiher E, Westoby M (2006). Rebuilding community ecology from functional traits. Trends Ecol. Evol..

[3] Kraft N (2014). Community assembly, coexistence, and the environmental filtering metaphor. Funct. Ecol..

[4] de la Sancha NU, Higgins CL, Presley SJ, Strauss RE (2014). Metacommunity structure in a highly fragmented forest: has deforestation in the Atlantic Forest altered historic biogeographic patterns?. Divers. Distrib..

[5] Leibold M, Mikkelson G (2002). Coherence, species turnover, and boundary clumping: Elements of meta-community structure. Oikos.

[6] Presley S, Higgins C, Willig M (2010). A comprehensive framework for the evaluation of metacommunity structure. Oikos.

[7] Dallas T, Drake JM (2014). Relative importance of environmental, geographic, and spatial variables on zooplankton metacommunities. Ecosphere.

[8] Heino J, Mykrä H, Muotka T (2009). Temporal variability of nestedness and idiosyncratic species in stream insect assemblages. Divers. Distrib..

[9] Henriques-Silva R, Lindo Z, Peres-Neto PR (2013). A community of metacommunities: exploring patterns in species distributions across large geographical areas. Ecology.

[10] Dallas T, Drake JM (2014). Relative importance of environmental, geographic, and spatial variables on zooplankton metacommunities. Ecosphere.

[11] Erős T (2014). Quantifying temporal variability in the metacommunity structure of stream fishes: The influence of non-native species and environmental drivers. Hydrobiologia.

[12] Fernandes IM, Henriques-Silva R, Penha J, Zuanon J, Peres-Neto PR (2014). Spatiotemporal dynamics in a seasonal metacommunity structure is predictable: The case of floodplain-fish communities. Ecography.

[13] Tonkin JD (2018). The role of dispersal in river network metacommunities: Patterns, processes, and pathways. Freshw. Biol..

[14] Kim S, Chung S, Park H, Cho Y, Lee H (2019). Analysis of environmental factors associated with cyanobacterial dominance after river weir installation. Water.

[15] Deng J (2014). Effects of nutrients, temperature and their interactions on spring phytoplankton community succession in Lake Taihu, China. PLoS ONE.

[16] Yang J, Jiang H, Liu W, Wang B (2018). Benthic algal community structures and their response to geographic distance and environmental variables in the Qinghai-Tibetan lakes with different salinity. Front. Microbiol..

[17] Zhou J (2018). Microbial community structure and associations during a marine dinoflagellate bloom. Front. Microbiol..

[18] RDevelopmentCoreTeam (2013). R: A Language and Environment for Statistical Computing.

[19] Baird RB (2017). Standard Methods for the Examination of Water and Wastewater.

[20] Liaw A, Wiener M (2002). Classification and regression by randomForest. R News.

[21] Cajo JFTB (1986). Canonical correspondence analysis: a new eigenvector technique for multivariate direct gradient analysis. Ecology.

[22] Tuomisto H (2010). A diversity of beta diversities: straightening up a concept gone awry. Part 2. Quantifying beta diversity and related phenomena. Ecography.

[23] Clements FE (1936). Nature and structure of the climax. J. Ecol..

[24] Kurthen AL (2020). Metacommunity structures of macroinvertebrates and diatoms in high mountain streams, Yunnan, China. Front. Ecol. Evol..

[25] Vannote RL, Minshall GW, Cummins KW, Sedell JR, Cushing CE (1980). The river continuum concept. Can. J. Fish. Aquat. Sci..

[26] López-González C, Presley SJ, Lozano A, Stevens RD, Higgins CL (2012). Metacommunity analysis of Mexican bats: environmentally mediated structure in an area of high geographic and environmental complexity. J. Biogeogr..

[27] Heino J, Soininen J, Alahuhta J, Lappalainen J, Virtanen R (2017). Metacommunity ecology meets biogeography: effects of geographical region, spatial dynamics and environmental filtering on community structure in aquatic organisms. Oecologia.

[28] Heino J, Alahuhta J (2015). Elements of regional beetle faunas: faunal variation and compositional breakpoints along climate, land cover and geographical gradients. J. Anim. Ecol..

[29] Mallin MA, McIver MR, Ensign SH, Cahoon LB (2004). Photosynthetic and heterotrophic impacts of nutrient loading to blackwater streams. Ecol. Appl..

[30] B-Béres V (2019). Autumn drought drives functional diversity of benthic diatom assemblages of continental intermittent streams. Adv. Water Resour..

[31] Kagalou I, Petridis D, Tsimarakis G (2003). Seasonal variation of water quality parameters and plankton in a shallow Greek lake. J. Freshw. Ecol..

[32] Padisák J, Crossetti LO, Naselli-Flores L (2009). Use and misuse in the application of the phytoplankton functional classification: a critical review with updates. Hydrobiologia.

[33] Schabhüttl S (2013). Temperature and species richness effects in phytoplankton communities. Oecologia.

[34] Chen S (2020). Geographical patterns of algal communities associated with different urban lakes in China. Int. J. Environ. Res. Public Health.

[35] Hwang S-J, Kim H-S, Shin J-K, Oh J-M, Kong D-S (2004). Grazing effects of a freshwater bivalve (*Corbicula leana* Prime) and large zooplankton on phytoplankton communities in two Korean lakes. Hydrobiologia.

[36] Moss B (2003). How important is climate? Effects of warming, nutrient addition and fish on phytoplankton in shallow lake microcosms. J. Appl. Ecol..

[37] Chen S (2019). Local habitat heterogeneity determines the differences in benthic diatom metacommunities between different urban river types. Sci. Total Environ..

