# Supplementary material for: Water quality drives the regional patterns of an algal metacommunity in interconnected lakes
Source: Sci Rep. 2021 Jun 30;11:13601. doi: 10.1038/s41598-021-93178-9 (PMC8245656; doi:10.1038/s41598-021-93178-9)
Supplement: Supplementary file 1 — Supplementary Information. [file 41598_2021_93178_MOESM1_ESM.docx]

**Water quality drives the regional patterns of an algal metacommunity in interconnected lakes**

Min Sung Kim^1,2^, Seok Hyun Ahn^1^, In Jae Jeong^1^, Tae Kwon Lee^1*^

^1^Department of Environmental Engineering, College of Health Science, Yonsei University, Gangwon 26493, South Korea

*Corresponding authors:

Tae Kwon Lee: tklee@yonsei.ac.kr

Supplementary Material

# Supplementary tables

**Supplementary Table S1. Season and annual variation of six lakes metacommunity structure**

|  | | PD | CP | UM | CC | SY | HC | ALL |
| --- | --- | --- | --- | --- | --- | --- | --- | --- |
| Season | Spring | C | C | C | QC | C | C | QC |
|  | Summer | QC | C | C | C | C | QC | C |
|  | Fall | QC | C | C | QC | C | QC | C |

* C, Clementsian; QC, Quasi-Clementsian

**Supplementary Table S2. Distribution of water qualities between six lakes.** The values indicated mean ± standard deviation of water qualities.

|  | PD | CP | UM | CC | SY | HC |
| --- | --- | --- | --- | --- | --- | --- |
| Temperature  (℃) | 17.5 ± 5.5^a^ | 17.1 ± 5.5^a^ | 12.4 ± 4.5^a^ | 17.2 ± 5.4^a^ | 10.2 ± 4.1^c^ | 16.1 ± 4.9^b^ |
| Conductivity | 94.9 ± 18.3^a^ | 99 ± 27.0^c^ | 105.2 ± 28.3^c^ | 161.9 ± 33.5^c^ | 75 ± 10.0^d^ | 92.3 ± 15.1^b^ |
| pH | 8.1 ± 0.6^b^ | 7.9 ± 0.5^b^ | 7.8 ± 0.5^a^ | 7.9 ± 0.4^a^ | 7.5 ± 0.7^c^ | 8.2 ± 0.5^b^ |
| BOD | 0.9 ± 0.4^a^ | 1.0 ± 0.4^b^ | 0.7 ± 0.4^a^ | 1.3 ± 0.4^b^ | 1.1 ± 0.3^a^ | 1.2 ± 0.4^c^ |
| COD | 2.7 ± 0.9^a^ | 3.3 ± 0.5^b^ | 2.2 ± 0.7^c^ | 3.9 ± 0.4^d^ | 2.1 ± 0.4^e^ | 3.1 ± 0.6^e^ |
| TN | 1.7 ± 0.6^a^ | 1.7 ± 0.4^b^ | 1.5 ± 0.4^a^ | 2.0 ± 0.4^b^ | 1.6 ± 0.3^bc^ | 2.0 ± 0.6^c^ |
| NH_4_^+^ | 0.08 ± 0.08^bc^ | 0.05 ± 0.07^cd^ | 0.07 ± 0.12^a^ | 0.05 ± 0.03^b^ | 0.04 ± 0.05^e^ | 0.09 ± 0.06^d^ |
| TP | 0.02 ± 0.03^a^ | 0.02 ± 0.02^b^ | 0.01 ± 0.01^b^ | 0.04 ± 0.02^c^ | 0.01 ± 0.01^b^ | 0.03 ± 0.04^d^ |
| PO_4_^-^ | 0.003 ± 0.004^a^ | 0.008 ± 0.009^a^ | 0.003 ± 0.003^b^ | 0.01 ± 0.01^bc^ | 0.002 ± 0.002^c^ | 0.004 ± 0.004^c^ |

* Different small letters indicate significant differences between values of water qualities in sampling sites.
** BOD, Biochemical oxygen demand; COD, Chemical oxygen demand; TN, Total nitrogen; NH_4_^+^, Ammonium; TP, Total phosphorus; PO_4_^-^, Phosphate (unit: mg/L)

**Supplementary Table S3. Distribution of seasonal precipitation of six lakes in sampling periods**

| Average  precipitation  per season (mm) | **Year** | **Season** | **PD** | **CP** | **UM** | **CC** | **SY** | **HC** |
| --- | --- | --- | --- | --- | --- | --- | --- | --- |
|  | **2008** | Spring | 36.7±25.6 | 43.7±37.8 | 59.0±27.9 | 55.7±23.7 | 42.3±41.1 | 51.0±21.1 |
|  |  | Summer | 380.0±157.1 | 373.3±178.8 | 330.7±166.5 | 331.7±266.6 | 349.0±161.3 | 318.0±158.1 |
|  |  | Fall | 52.7±40.0 | 40.7±26.6 | 33.3±21.5 | 23.7±9.4 | 37.7±25.0 | 40.0±25.9 |
|  | **2009** | Spring | 65.7±27.0 | 55.7±36.1 | 67.7±16.5 | 79.7±12.8 | 58.3±31.8 | 56.3±18.6 |
|  |  | Summer | 139.0±94.1 | 404.3±257.1 | 337.7±200.2 | 335.3±177.8 | 344.7±207.1 | 301.3±75.3 |
|  |  | Fall | 42.3±2.6 | 51.3±8.8 | 29.0±12.0 | 33.3±13.7 | 37.3±9.0 | 28.3±11.1 |
|  | **2010** | Spring | 85.0±25.0 | 119.0±32.0 | 72.0±19.0 | 89.0±23.0 | 81.5±22.5 | 62.0±13.0 |
|  |  | Summer | 257.0±101.1 | 318.3±133.3 | 227.0±156.7 | 291.0±147.3 | 240.3±171.8 | 229.7±123.2 |
|  |  | Fall | 198.7±250.9 | 217.3±280.0 | 158.0±197.4 | 149.3±178.1 | 163.3±202.1 | 128.0±155.8 |
|  | **2011** | Spring | 82.5±31.5 | 91.5±19.5 | 100.5±3.5 | 101.5±3.5 | 129.5±14.5 | 90.5±2.5 |
|  |  | Summer | 587.3±373.6 | 534.0±328.0 | 510.3±320.4 | 500.3±341.7 | 520.0±323.0 | 391.0±248.5 |
|  |  | Fall | 46.7±2.5 | 54.3±19.1 | 46.3±18.2 | 56.3±31.5 | 50.7±20.3 | 46.3±21.4 |
|  | **2012** | Spring | 65.7±50.3 | 87.3±70.2 | 70.3±39.8 | 76.7±57.7 | 73.0±48.1 | 45.0±34.0 |
|  |  | Summer | 374.0±186.1 | 335.0±152.1 | 221.3±87.8 | 257.0±103.5 | 243.7±90.4 | 179.3±83.2 |
|  |  | Fall | 130.5±55.5 | 138.5±36.5 | 89.0±19.0 | 115.5±28.5 | 103.5±29.5 | 88.0±28.0 |
|  | **2013** | Spring | 102.5±29.5 | 112.5±26.5 | 101.5±45.5 | 101.0±17.0 | 77.0±18.0 | 56.5±11.5 |
|  |  | Summer | 396.7±358.5 | 115.0±54.0 | 349.3±313.1 | 362.0±358.6 | 395.3±384.7 | 327.3±317.6 |
|  |  | Fall | 94.0±90.0 | 53.3±32.5 | 71.3±63.8 | 72.3±57.2 | 72.7±58.7 | 70.0±54.4 |
|  | **2015** | Spring | 40.7±30.6 | 52.7±40.0 | 43.7±35.8 | 46.7±34.6 | 44.3±34.8 | 36.3±25.8 |
|  |  | Summer | 139.7±58.9 | 147.0±125.2 | 99.3±86.8 | 153.3±134.9 | 129.0±93.4 | 111.0±98.3 |
|  |  | Fall | 57.3±38.2 | 56.3±38.2 | 55.7±43.3 | 76.0±64.9 | 65.0±49.3 | 57.0±42.9 |
|  | **2016** | Spring | 84.7±38.1 | 89.7±40.7 | 78.7±23.0 | 1181.0±22.0 | 77.3±23.3 | 70.0±25.8 |
|  |  | Summer | 166.0±140.1 | 200.3±212.0 | 232.0±238.6 | 199.0±222.1 | 222.7±238.6 | 147.7±150.9 |
|  |  | Fall | 44.3±24.2 | 41.0±24.2 | 58.7±36.1 | 72.5±29.5 | 59.7±39.7 | 44.3±33.4 |

**Supplementary Table S4. Climate information of six lakes within sampling periods**

| Temperature  (℃) | **Year** | **Season** | **PD** | **CP** | **UM** | **CC** | **SY** | **HC** |
| --- | --- | --- | --- | --- | --- | --- | --- | --- |
|  | **2008** | Spring | 11.6±4.6 | 11.6±4.6 | 11.6±4.7 | 11.6±4.7 | 11.6±4.7 | 10.8±4.8 |
|  |  | Summer | 23.8±1.9 | 23.8±1.9 | 23.2±2.0 | 23.2±2.0 | 23.2±2.0 | 22.6±1.8 |
|  |  | Fall | 13.5±6.1 | 13.5±6.1 | 12.8±6.0 | 12.8±6.0 | 12.8±6.0 | 12.2±6.1 |
|  | **2009** | Spring | 12.2±5.1 | 12.2±5.1 | 11.8±5.2 | 11.8±5.2 | 11.8±5.2 | 10.6±5.4 |
|  |  | Summer | 23.6±1.0 | 23.6±1.0 | 23.1±1.2 | 23.1±1.2 | 23.1±1.2 | 22.3±1.3 |
|  |  | Fall | 13.2±5.8 | 13.2±5.8 | 12.8±5.8 | 12.8±5.8 | 12.8±5.8 | 11.8±5.9 |
|  | **2010** | Spring | 13.5±3.9 | 13.5±3.9 | 13.2±4.0 | 13.2±4.0 | 13.2±4.0 | 12.0±4.2 |
|  |  | Summer | 24.9±1.5 | 24.9±1.5 | 24.8±1.4 | 24.8±1.4 | 24.8±1.4 | 24.1±1.4 |
|  |  | Fall | 13.0±6.6 | 13.0±6.6 | 12.4±6.6 | 12.4±6.6 | 12.4±6.6 | 11.7±6.7 |
|  | **2011** | Spring | 9.0±1.6 | 9.0±1.6 | 13.6±3.5 | 13.6±3.5 | 13.6±3.5 | 12.9±3.8 |
|  |  | Summer | 24.1±1.4 | 24.1±1.4 | 23.8±1.4 | 23.8±1.4 | 23.8±1.4 | 23.2±1.4 |
|  |  | Fall | 14.0±4.6 | 14.0±4.6 | 13.2±4.8 | 13.2±4.8 | 13.2±4.8 | 12.5±4.7 |
|  | **2012** | Spring | 12.0±5.5 | 12.0±5.5 | 11.3±5.8 | 11.3±5.8 | 11.3±5.8 | 10.3±6.0 |
|  |  | Summer | 25.2±1.1 | 25.2±1.1 | 24.7±1.2 | 24.7±1.2 | 24.7±1.2 | 23.6±1.1 |
|  |  | Fall | 16.2±3.3 | 16.2±3.3 | 15.9±3.3 | 15.9±3.3 | 15.9±3.3 | 15.2±3.1 |
|  | **2013** | Spring | 13.9±4.0 | 13.9±4.0 | 13.5±4.4 | 13.5±4.4 | 13.5±4.4 | 12.5±4.4 |
|  |  | Summer | 25.2±1.1 | 25.2±1.1 | 25.3±1.2 | 25.3±1.2 | 25.3±1.2 | 24.4±1.2 |
|  |  | Fall | 13.2±6.4 | 13.2±6.4 | 12.9±6.5 | 12.9±6.5 | 12.9±6.5 | 11.5±6.6 |
|  | **2015** | Spring | 12.6±5.3 | 12.6±5.3 | 12.3±5.6 | 12.3±5.6 | 12.3±5.6 | 11.0±5.6 |
|  |  | Summer | 24.7±1.1 | 24.7±1.1 | 24.7±0.9 | 24.7±0.9 | 24.7±0.9 | 23.2±1.1 |
|  |  | Fall | 14.2±4.9 | 14.2±4.9 | 14.0±5.1 | 14.0±5.1 | 14.0±5.1 | 12.6±5.0 |
|  | **2016** | Spring | 13.2±5.1 | 13.2±5.1 | 12.9±5.4 | 12.9±5.4 | 12.9±5.4 | 11.7±5.3 |
|  |  | Summer | 25.3±1.4 | 25.3±1.4 | 25.0±1.3 | 25.0±1.3 | 25.0±1.3 | 23.8±1.3 |
|  |  | Fall | 14.3±6.4 | 14.3±6.4 | 13.7±6.5 | 13.7±6.5 | 13.7±6.5 | 12.6±6.5 |

**Supplementary Table S5. The loading of first axis canonical correspondence analysis (CCA)** The value of table means loading value from CCA 1.

|  | ALL | PD | CP | UM | CC | SY | HC |
| --- | --- | --- | --- | --- | --- | --- | --- |
| Temperature | -0.061 | **-0.844** | **0.703** | **0.732** | **0.734** | **-0.781** | 0.205 |
| Conductivity | **0.743** | 0.274 | -0.259 | -0.3 | -0.217 | -0.326 | **0.885** |
| pH | 0.354 | **0.741** | **-0.623** | 0.199 | 0.176 | -0.279 | -0.229 |
| BOD | **0.57** | **0.823** | **-0.816** | **-0.592** | 0.187 | 0.297 | -0.29 |
| COD | **0.711** | 0.311 | -0.388 | 0.238 | 0.129 | **-0.524** | 0.046 |
| TN | 0.355 | 0.269 | -0.079 | -0.321 | 0.091 | **0.52** | -0.26 |
| NH_4_^+^ | 0.072 | 0.095 | -0.056 | -0.305 | -0.155 | -0.367 | -0.066 |
| TP | 0.189 | -0.293 | 0.19 | 0.057 | 0.298 | -0.452 | 0.061 |
| PO­­_4_^-^ | 0.012 | -0.369 | 0.085 | -0.269 | 0.332 | -0.208 | 0.133 |

*The bold text was indicated that loading value was higher than 0.5
**BOD, Biochemical oxygen demand; COD, Chemical oxygen demand; TN, Total nitrogen; NH_4_^+^, Ammonium; TP, Total phosphorus; PO_4_^-^, Phosphate

# Supplementary figures

**
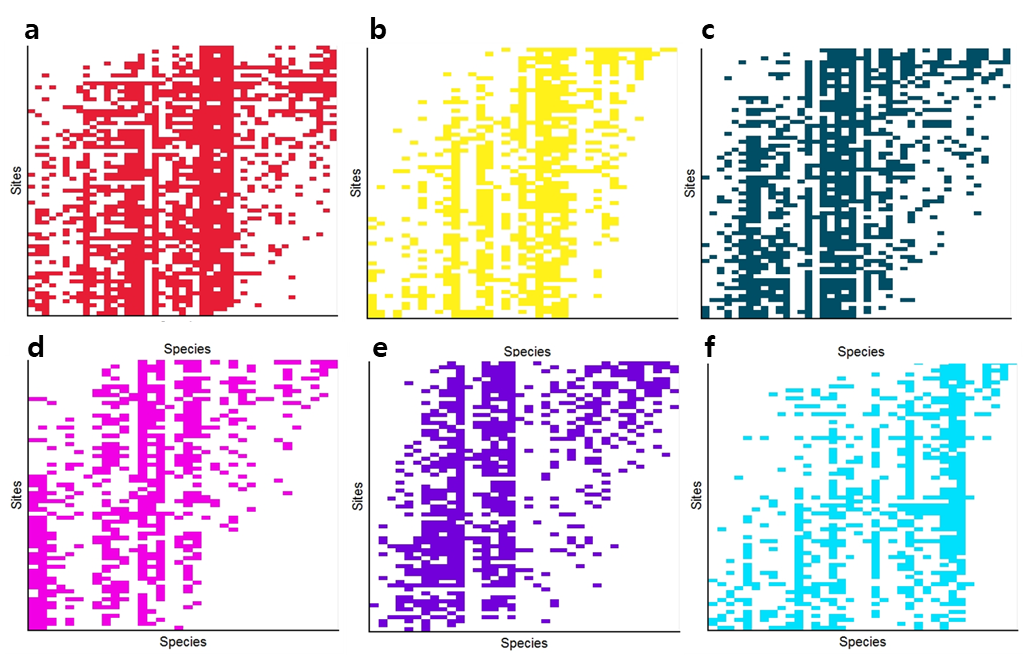
**

**Figure S1.** **Metacommunity structures of sampling sites (a) Paldang (PD), (b) Cheongpyeong (CP), (c) Uiam (UM), (d) Chuncheon (CC), (e) Soyang (SY) and (f) Hwacheon (HC).** All metacommunity structures classified as Clemensian metacommunity. Only Paldang was defined as quasi-Clemensian.

**
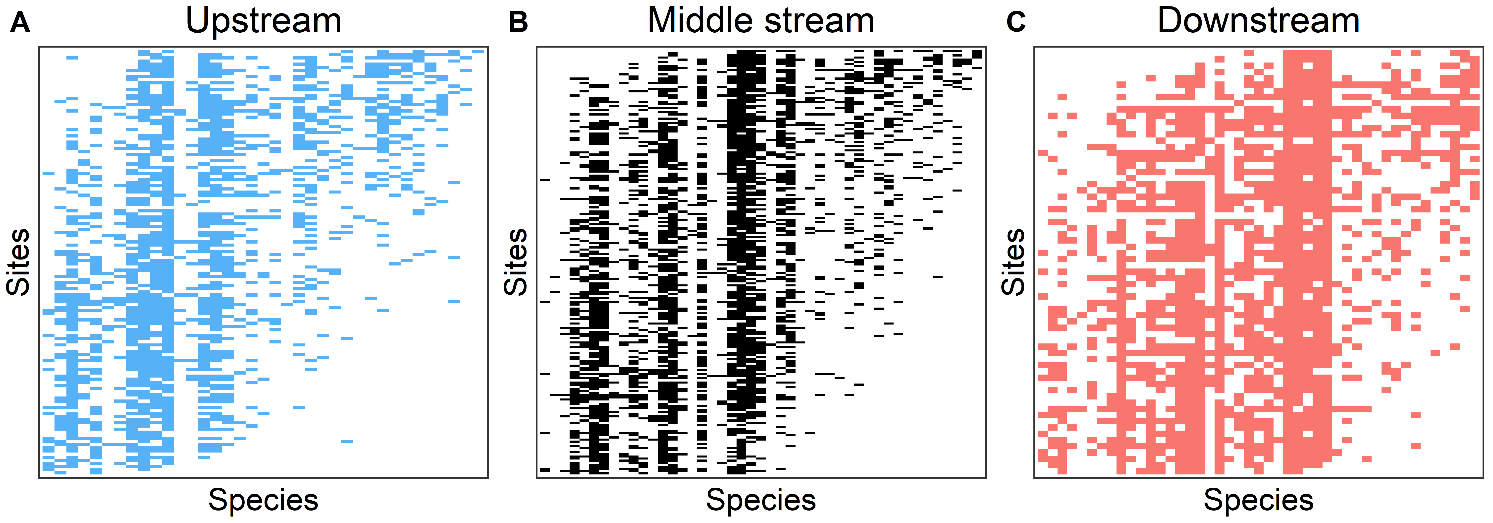
**

**Figure S2.** **Visualization of metacommunity structures by (a) upstream, (b) midstream and (c) downstream.** For the upstream and midstream represents Clementsian structure that algal community gradually shifted by sites but downstream showed qausi-Clementsian.
